# Tryptophan hydroxylase 1 drives glioma progression by modulating the serotonin/L1CAM/NF-κB signaling pathway

**DOI:** 10.1186/s12885-022-09569-2

**Published:** 2022-04-26

**Authors:** Jie Zhang, Zhangchao Guo, Qiangli Xie, Chuanhong Zhong, Xiangyu Gao, Qiumei Yang

**Affiliations:** 1 Department of Neurosurgery, Ya’ an people’s Hospital, Ya’ an, 625000 People’s Republic of China; 2 Department of Neurosurgery, Affiliated Hospital of Southwest Medical University, Luzhou, People’s Republic of China; 3 Department of Cardiology, Chengdu Qingbaijiang District People’s Hospital, Chengdu, People’s Republic of China; 4 Pediatrics of Ya’ an people’s Hospital, Ya’ an, People’s Republic of China; 5 Department of Geriatrics, Luzhou people’s Hospital, Luzhou, People’s Republic of China

**Keywords:** TPH-1, Glioma, Serotonin, L1CAM

## Abstract

**Background:**

Glioma is one of the main causes of cancer-related mortality worldwide and is associated with high heterogeneity. However, the key players determining the fate of glioma remain obscure. In the present study, we shed light on tumor metabolism and aimed to investigate the role of tryptophan hydroxylase 1 (TPH-1) in the advancement of glioma.

**Method:**

Herein, the levels of TPH-1 expression in glioma tissues were evaluated using The Cancer Genome Atlas (TCGA) database. Further, the proliferative characteristics and migration ability of TPH-1 overexpressing LN229/T98G cells were evaluated. Additionally, we performed a cytotoxicity analysis using temozolomide (TMZ) in these cells. We also examined the tumor growth and survival time in a mouse model of glioma treated with chemotherapeutic agents and a TPH-1 inhibitor.

**Results:**

The results of both clinical and experimental data showed that excess TPH-1 expression resulted in sustained glioma progression and a dismal overall survival in these patients. Mechanistically, TPH-1 increased the production of serotonin in glioma cells. The elevated serotonin levels then augmented the NF-κB signaling pathway through the upregulation of the L1-cell adhesion molecule (L1CAM), thereby contributing to cellular proliferation, invasive migration, and drug resistance. In vivo experiments demonstrated potent antitumor effects, which benefited further from the synergistic combination of TMZ and LX-1031.

**Conclusion:**

Taken together, these data suggested that TPH-1 facilitated cellular proliferation, migration, and chemoresistance in glioma through the serotonin/L1CAM/NF-κB pathway. By demonstrating the link of amino acid metabolic enzymes with tumor development, our findings may provide a potentially viable target for therapeutic manipulation aimed at eradicating glioma.

**Supplementary Information:**

The online version contains supplementary material available at 10.1186/s12885-022-09569-2.

## Background

Glioma is among the tumor types that show the worst prognoses and is characterized by low differentiation, extensive angiogenesis, and a high invasive potential [1]. The mainstay of glioma treatment consists of surgical resection alone or in combination with chemotherapy and radiation therapy, however, the efficacy remains modest [2]. Tumor relapse and the development of chemoresistance represent the predominant cause of treatment failure, leading to a dismal five-year survival rate of only 5% and a median survival duration of < 15 months [3, 4]. To date, the molecular mechanisms underlying glioma development remain unidentified. Thus, further investigations are warranted to gain insight into glioma progression and provide a new avenue for tumor elimination.

Tumor development is a complicated and multistage process that involves both genetic and environmental factors [5]. Cellular metabolism is extensively reported to function as a master regulator of tumor behaviors [6]. To cope with the high proliferation rate, cancer cells may undergo metabolic reprogramming and utilize high glucose for sufficient ATP supply. Recent findings indicate that in addition to an abundance of glucose, amino acids are exploited by tumors to fulfill the requirements of energy and biosynthesis in cells [7]. Tryptophan, an essential amino acid, serves a significant role in malignant conversion and tumor advancement [8]. The majority of Trp degradation occurs through the kynurenine (Kyn) pathway, resulting in the generation of several metabolites with diverse biological functions. The rate-limiting step of the Kyn pathway is catalyzed by indoleamine-2, 3-dioxygenase (IDO), and tryptophan-2, 3-dioxygenase (TDO), which are intimately linked to the modulation of immune responses and offer promising targets for cancer therapy [9]. While a small fraction of Trp is converted to 5-hydroxytryptophan by tryptophan hydroxylase-1 (TPH-1) and provides precursors for the production of serotonin, a classical neurotransmitter with multiple roles in the cardiovascular system, endocrinology, gut motility, reproductive function, and carcinogenesis [10, 11]. In fact, TPH-1 is up-regulated in several tumor types [12]. Jaya et al. report that TPH-1 is preferentially expressed in triple-negative breast cancer (TNBC) and its silencing markedly suppresses cellular proliferation and invasion [13]. They further suggest that TPH-1 facilitates tumor progression via autocrine serotonin signaling. Moreover, TPH-1 knockdown or 4-chloro-DL-phenylalanine (a TPH-1 inhibitor) treatment can retard tumor growth in mice models of colorectal cancer [14]. At present, telotristat, a tryptophan hydroxylase inhibitor, is being evaluated in clinical trials for the treatment of patients with metastatic neuroendocrine tumors [15]. Despite the substantial research efforts, a clear understanding of the role of TPH-1 in tumor progression is lacking.

The NF-κB transcription factor plays an essential role in inflammation and innate immunity [16, 17]. More importantly, recent studies suggest that NF-κB serves as a crucial player in different steps of tumor progression [18]. NF-κB cooperates with multiple signaling molecules and pathways, such as STAT3, P53, or the adhesion molecule, LCAM. The crosstalk between NF-κB and pro-survival signals can also be mediated by different kinases, including AKT and P38, which facilitate NF-κB transcriptional activity or affect upstream signaling [19, 20]. Additionally, many of the genes transcribed by NF-κB can promote carcinogenesis, including IL-1β, BCL2, and VEGF [21, 22]. However, the correlation between tryptophan metabolism and NF-κB during tumor progression remains poorly understood.

Using The Cancer Genome Atlas (TCGA) database, we observed that TPH-1 expression was markedly augmented in glioma tissues and that patients with a high TPH-1 expression exhibited poorer overall survival rates. In addition, data from the in vitro experiments showed that TPH-1 facilitated glioma cell proliferation and decreased the efficacy of chemotherapy by catalyzing the production of serotonin. Our study further indicated that TPH-1 drove glioma development in an L1-cell adhesion molecule (L1-CAM)/NF-κB dependent manner. Importantly, a combination of chemotherapeutic drugs and a TPH-1 inhibitor yielded excellent treatment outcomes in the xenograft mouse models. These results are expected to motivate extensive research for targeting TPH-1 signals in pursuit of optimal therapeutic strategies for glioma.

## Materials and methods

### Cell culture and reagents

LN229 and T98G cell lines were purchased from American Type Culture Collection (ATCC) and maintained in RPMI 1640 complete culture medium (Gibco, USA) supplemented with 10% fetal bovine serum (FBS, Gibco, USA). TPH-1 overexpressing LN229 and T98G cells were generated by Cyagen (China) and the expressions were determined by western blotting. Temozolomide (TMZ) and serotonin were purchased from Sigma (USA). The NF-κB inhibitor, QNZ (EVP4593), was obtained from Selleck (USA). TPH-1 inhibitor, LX-1031, was obtained from Abcam (UK).

### Cellular proliferation assay

Cellular proliferation in LN229 and T98G cells was determined using the Cell Counting Kit-8 (CCK8) Assay Kit (Solarbio, China). Briefly, 2 × 10^3^ tumor cells were resuspended and seeded per well in a 96-well plate. The cell numbers were monitored daily using the CCK-8 solution. A microplate reader (Thermo Fisher, USA) was used to measure the sample absorbance (OD) at 450 nm. Three independent experiments were performed.

### Transwell assay

1 × 10^5^ T98G or 5 × 10^4^ LN229 cells were seeded in a Transwell insert (8 μm, Corning, USA) for assessing the cellular migration ability. After 48 h, the migrating cells were fixed with paraformaldehyde and stained using crystal violet. The migrated cells were counted and each experiment was performed thrice independently.

### Clinical specimens

Paraffin sections of human glioma tissues were collected from the pathology department of Ya’ an People’s Hospital. Glioma tissues were divided into the TPH-1^high^ and TPH-1^low^ groups based on the median expression of TPH-1, which was determined by immunohistochemistry or immunofluorescence assays. All glioma patients were informed and provided written consent to participate in the study. The clinical experiments were performed according to the guidelines in the Declaration of Helsinki and approved by the Ethics Committee of Ya’ an People’s Hospital.

### Transcription and survival analysis in patients

Patient information, including clinical and gene-expression data, was obtained from TCGA database, which included data for 99 glioma patients (survival and RNA expression data, https://www.cbioportal.org/), 5 normal tissue and 156 glioma tissues (TPH1 RNA expression data, http://ualcan.path.uab.edu/index.html). Overall survival in the two groups were analyzed and compared by the Kaplan–Meier method. Differences in gene expression were tested for statistical significance by the Student’s t-test using GraphPad Prism software. Gene analysis were performed with the use of the open-source R software (2.1.0).

### RNA interference

For L1CAM silencing, LN229 and T98G cells were treated using siRNA oligonucleotides at a concentration of 100 nM using Oligofectamine (Thermo, USA). The siRNA sequences used were as follows: 5′-AGGGAUGGUGUCCACUUCAAATT-3 and 5′-UGAAGUCGAGCGAUCCGUAG-3′. The silencing efficiencies for LICAM in LN229 and T98G cells were examined by real-time PCR.

### Western blotting

Glioma cells were lysed using 1% NP40 buffer containing a protease inhibitor cocktail (Solarbio, China). The Protein Quantitative Analysis Kit (Solarbio, China) was used for protein quantification. 25 μg protein were separated using a 10% SDS gel and the separated proteins were transferred onto polyvinylidene fluoride immobilon-membranes. Subsequently, cropped (or not) immobilon-membranes were blocked using 5% nonfat dried milk and incubated with the following primary antibodies: anti-TPH-1 (Abcam, UK), anti-L1CAM (Abcam, UK), anti-NF-κB (Abcam, UK), and anti-β-actin (Abcam, UK). Next, the membranes were incubated using an HRP-conjugated secondary antibody (Abcam, UK) for one hour at room temperature. Proteins were visualized using the ECL detection kit (Thermo Fisher, USA).

### Immunofluorescence assay

Paraffin sections of glioma tissues were de-waxed and treated with sodium citrate for antigen retrieval. Subsequently, the samples were blocked with 5% bovine serum albumin for 30 min at room temperature, and incubated with the following primary antibodies: anti-TPH-1 (Abcam, UK), anti- serotonin antibody (Abcam, UK), and anti-L1CAM (Abcam, UK), and anti-NF-κB (Abcam, UK), overnight at 4 °C. These samples were then incubated with goat anti-rabbit secondary antibody (Abcam, UK) and the nuclei were stained with DAPI (Solarbio, China). The intensity of protein expression was analyzed using the Image J 6.0 software (USA).

### Enzyme-linked immunosorbent assay (Elisa) assay

Elisa assay was performed according to the guidance of manufacturer. Human Tryptophan Elisa Kits were purchased from Guidechem (China). Human Serotonin Elisa Kits were purchased from Abcam (UK). Three independent experiments were performed.

### Cytotoxicity assay

Cell apoptosis of LN229/T98G cells induced by TMZ or inhibitors was determined using the FITC-Annexin V/PE-PI apoptosis detection kit (Becton Dickinson, USA) according to the guidance of manufacturer. Briefly, LN229 and T98G cells were treated with TMZ (1 μg/ml) for 48 h. Then cells were stained with FITC-Annexin V/PE-PI staining solution for 20 min at room temperature. After that, cell apoptosis was detected by a C6 flow cytometer (Becton Dickinson, USA).

### TUNEL assay

Cell apoptosis in tumor tissues was determined by terminal deoxynucleotidyl transferase-mediated dUTP nick end-labeling (TUNEL) staining (Solarbio, China). Briefly, mice were injected with subcutaneously 1 × 10^6^ vector or TPH1 overexpressing LN229 cells on day 0. After 12 days, mice were treated with serotonin (1 μg in 100 ml PBS) or PBS by intratumor injection. On day 13, mice were treated with TMZ (0.05 μg in 100 ml PBS). On day 15, tumor tissues were harvested and frozen sections were permeabilized with 0.1% Triton X-100, and incubated with the TUNEL reaction mixture according to guidance of Kit. The percentage of apoptotic cells was determined as TUNEL positive cells/total number of cells.

### Animal protocols

Female 6 ~ 8 weeks old NOD-SCID mice were purchased from Huafukang (China) and maintained in Specific Pathogen Free (SPF) room. For tumor volume assay, mice were subcutaneously injected with 10^6^ LN229 cells (*n* = 6 in each group). After 10 days, mice were treated with PBS, TMZ (5 mg/kg), LX-1031(5 mg/kg) or combining therapy twice a week. Tumor volume and survival of mice was recorded every day. The calculation formula of tumor volume is: tumor volume = length × width ^2^/2. The animal studies were conducted in accordance with the Public Health Service Policy and complied with the WHO guidelines for the humane use and care of animals. All animal protocols were monitored by the Animal Ethics Committee of Ya’ an people’s Hospital.

### Statistical analysis

All data were presented as the mean ± SEM and analyzed using GraphPad 7.0. The statistical significance between the two groups was calculated using the Student’s t-test or a one-way ANOVA for three or more groups. Kaplan–Meier curves were used for survival analysis. All experiments in our study were performed in independent triplicates. *, *p* < 0.05; **, *p* < 0.01; n.s, no significant difference.

## Results

### TPH-1 promotes glioma progression and correlates with a poor progression

**Fig. 1:**
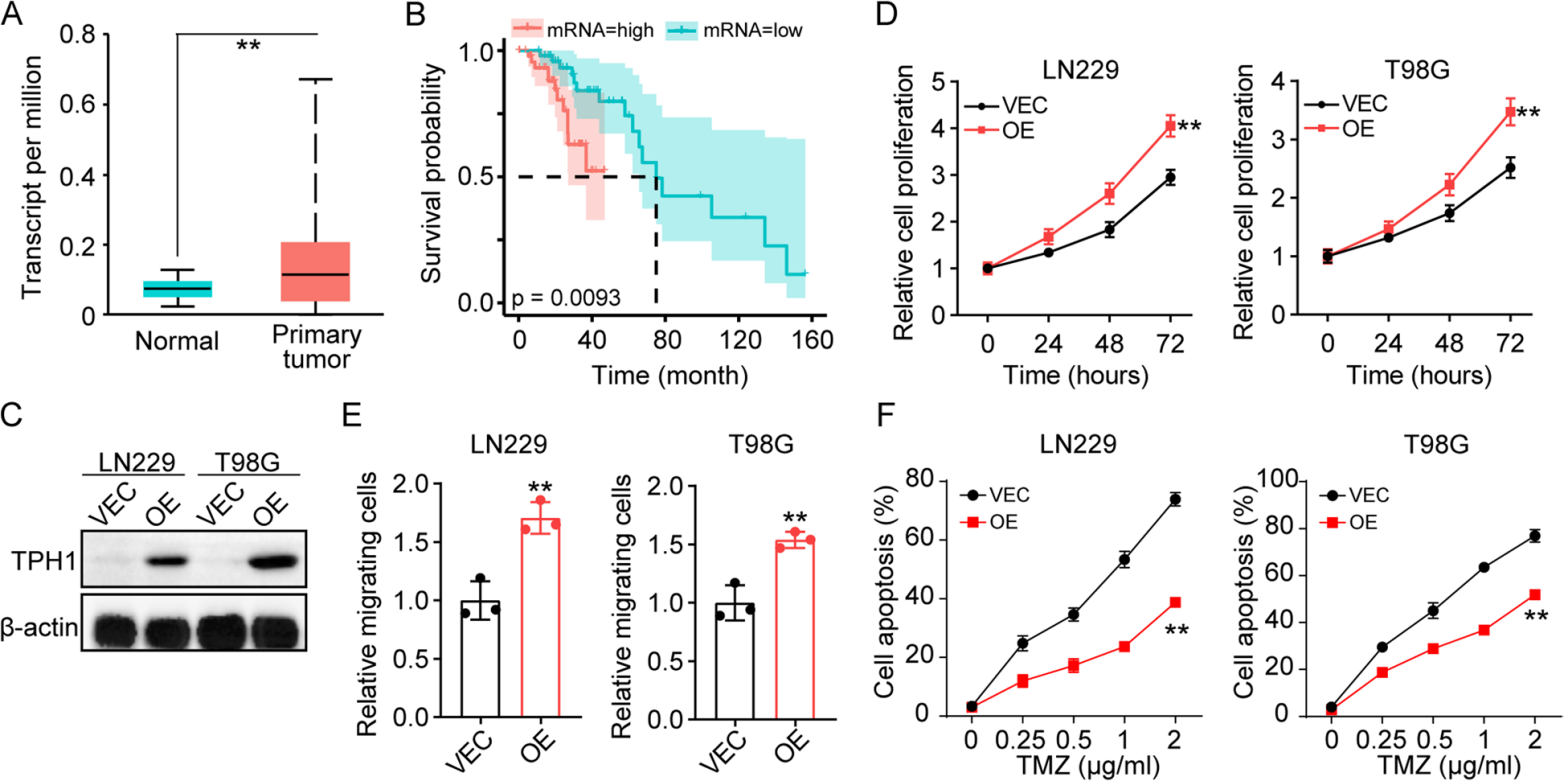
TPH-1 promoted glioma progression. **A** the transcriptome expression of TPH-1 in 156 glioma tissues and 5 normal tissues. **B** overall survival of glioma patients divided into high *TPH-1* (*n* = 49) and low *TPH-1* (*n* = 50) groups. **C** western blotting of TPH-1 in LN229/T98G (VEC) and TPH-1 overexpressing (OE) LN229/T98G cells. **D** cell proliferation of LN229/T98G (VEC) and TPH-1 overexpressing LN229/T98G cells (OE) determined by CCK8 assay. **E** relative migrating cells of LN229/T98G (VEC) and TPH-1 overexpressing LN229/T98G cells (OE) determined by transwell assay. **F** cell apoptosis of LN229/T98G (VEC) and TPH-1 overexpressing LN229/T98G cells (OE) treated with TMZ (48 h)

To assess the potential relevance of TPH-1 in glioma development, first, we evaluated the transcriptomic expression of TPH-1 in 156 glioma tissues and compared them to those in the normal tissues using TCGA database. Intriguingly, an elevated expression of TPH-1 was observed in glioma tissues relative to the normal tissues (Fig. 1A), suggesting the potential role of TPH-1 in promoting glioma development. To validate our results, we evaluated the overall survival of glioma patients. The top 10% of the patients with the highest TPH-1 expression were categorized into the high TPH-1 group (*n* = 49) and those with the lowest 10% expression were divided into the low TPH-1 group (*n* = 50). Indeed, patients with a high TPH-1 expression exhibited a poor overall survival relative to those in the low TPH-1 group (Fig. 1B). Given the poor prognosis of glioma patients with a high TPH-1 expression, we reasonably speculated whether TPH-1 expression could influence the cellular proliferation/migration in glioma. Thus, TPH-1 was overexpressed in glioma cancer cell lines, LN229 and T98G (Fig. 1C). The cellular proliferation/migration abilities were determined using the CCK8 and Transwell assays. Notably, TPH-1 overexpression significantly promoted cellular proliferation (Fig. 1D) and migration (Fig. 1E) of glioma cells. In addition, TPH-1 overexpressing LN229/T98G cells exhibited enhanced resistance to the chemotherapeutic agent, TMZ (Fig. 1F), suggesting that a high level of TPH-1 resulted in enhanced tumor growth and reduced chemo-sensitivity in glioma. To further confirm the role of TPH1 in regulating the responses to TMZ, mice were subcutaneously injected with 1 × 10^6^ vector or TPH1 overexpressing LN229 cells, and treated with TMZ on day 13. A TUNEL assay was performed to evaluate the cellular cytotoxicity in vivo. Indeed, TPH1 overexpression suppressed the cytotoxicity of TMZ in vivo (Fig. S1A). Taken together, these results implied that TPH-1 played a role in promoting glioma progression and was correlated with poor progression.

### Serotonin derived from tryptophan hydroxylation drives proliferation and invasion of glioma cells

**Fig. 2:**
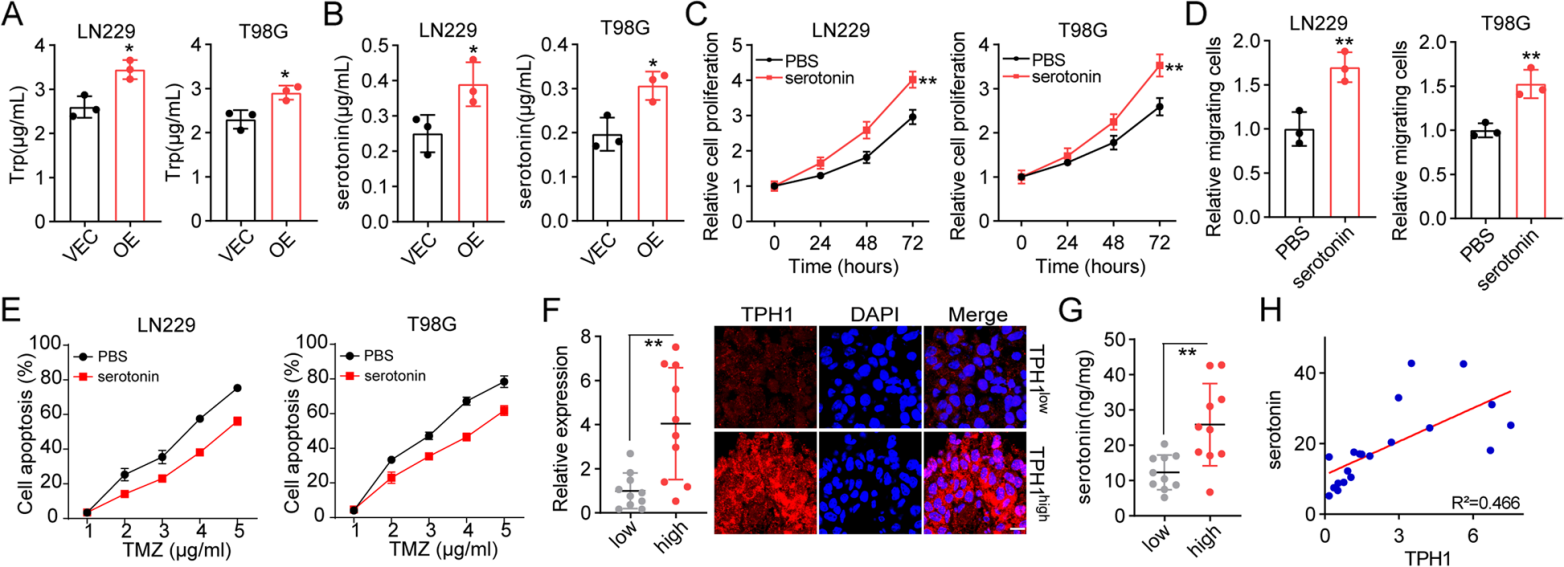
Serotonin drove glioma cells proliferation and invasion. **A** and **B** 10^5^ LN229/T98G (VEC) or TPH-1 overexpressing LN229/T98G cells (OE) were cultured in DMEM culture medium for 48 h. Trp consumption (**A**) and serotonin production (**B**) was determined by Elisa assay. **C** cell proliferation of LN229/T98G cells treated with PBS or serotonin (10 nM). **D** relative migrating cells of LN229/T98G cells treated with PBS or serotonin (10 nM). **E** LN229 or T98G cells were pre-treated with PBS or serotonin (10 nM) for 48 h. Then cell apoptosis of LN229/T98G treated with PBS or TMZ (48 h) were determined. **F** immunofluorescence staining of TPH-1 in TPH-1^high^ and TPH-1^low^ glioma tissues from patients. The scale bar was 25 μm. **G** serotonin secretion in TPH-1^high^ and TPH-1^low^ glioma tissues from patients, detected by Elisa analysis. **H** the correlation analysis between TPH-1 and serotonin in 20 glioma tissues (R^2^ = 0.466)

We aimed to elucidate the mechanism underlying TPH-1-induced glioma progression. TPH-1 is reportedly involved in amino acid metabolism and can catalyze the hydroxylation of L-Trp to serotonin. Recent studies suggest that serotonin can promote cell proliferation or invasion in breast/liver cancers [11]. This led us to reasonably speculate that TPH-1 may mediate the Trp hydroxylation to produce serotonin, thereby promoting glioma development. To confirm our hypothesis, we cultured tumor cells for 48 h, and examined the Trp consumption and serotonin production using the supernatant. Enhanced Trp consumption (Fig. 2A) and serotonin production (Fig. 2B) were observed in TPH-1 overexpressing LN229/T98G cells relative to LN229/T98G cells, suggesting that TPH-1 could catalyze the hydroxylation of L-Trp to generate serotonin. Subsequently, we treated LN229/T98G cells with serotonin and evaluated their proliferation/migration abilities. Serotonin treatment significantly strengthened the cellular proliferative characteristics (Fig. 2C) and migration phenotypes (Fig. 2D) of glioma cells. Moreover, serotonin treatment enhanced the TMZ resistance both in vitro and in vivo (Fig. 2E and S1B). These results suggested that TPH-1 mediated serotonin production could promote glioma progression. In an attempt to further confirm the correlation between TPH-1 expression and serotonin production in vivo, we collected 20 tumor tissues from glioma patients and determined the expression of TPH-1 and serotonin production by immunofluorescence and ELISA. The intensity of TPH-1 expression was quantified using the Image-J software. Increased serotonin levels were observed in TPH-1^high^ glioma tissues relative to the TPH-1^low^ glioma tissues (Fig. 2F and G). The correlational analysis between serotonin production and TPH-1 expression in Fig. 2F and G was performed in glioma tissues, wherein serotonin was found to be positively correlated with THP1 (Fig. 2H). Collectively, these findings highlighted that TPH-1 mediated tryptophan hydroxylation could produce serotonin, thereby promoting glioma progression.

### Serotonin upregulates L1CAM signaling to regulate glioma progression

**Fig. 3:**
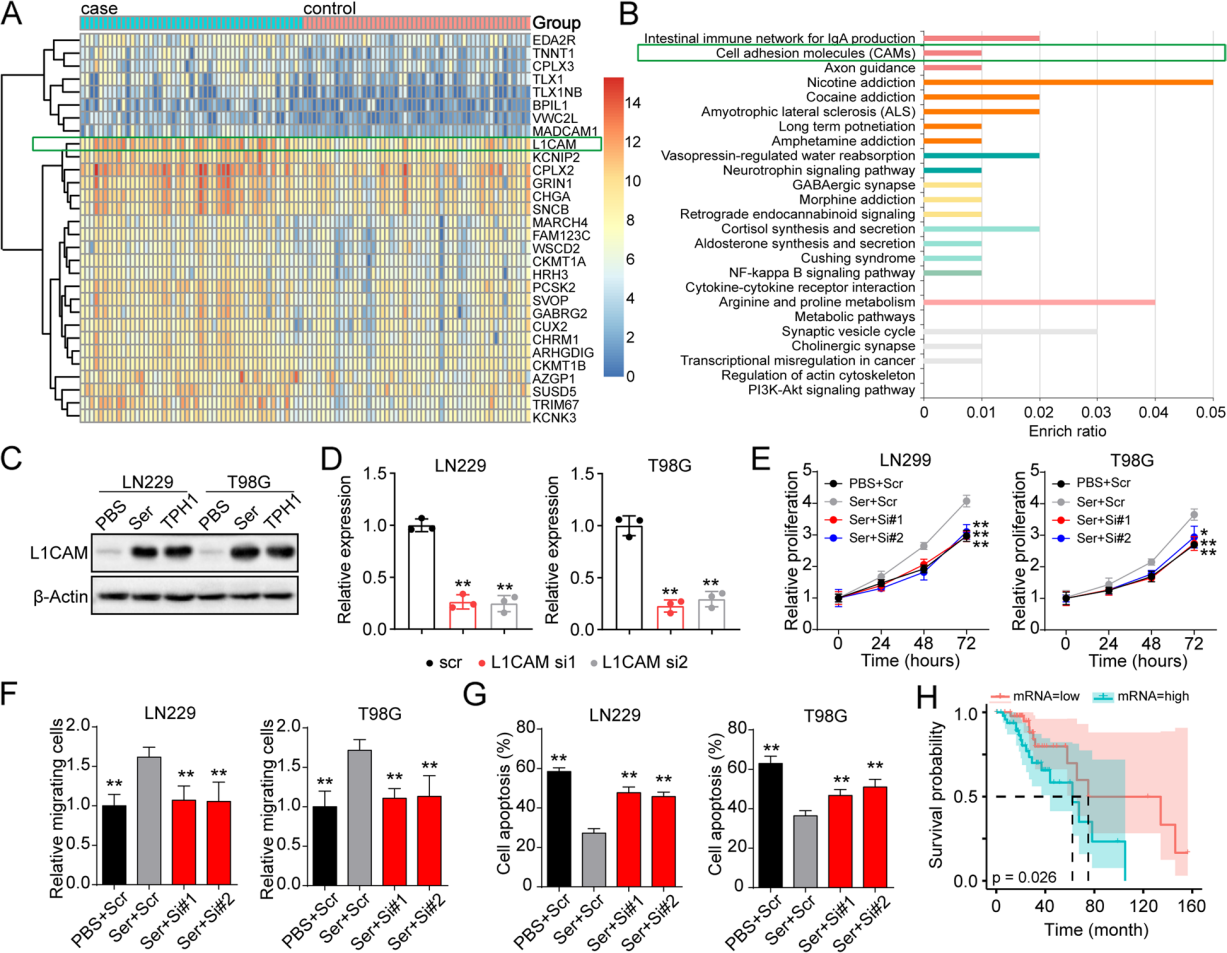
Serotonin upregulated L1CAM signaling to regulate glioma progression. **A** Heatmap of top 30 overexpressing genes in TPH-1^high^ glioma patients (*n* = 49) in comparison with TPH-1^low^ glioma patients (*n* = 50). **B** GO enrichment analysis in TPH-1^high^ glioma patients (*n* = 49) in comparison with TPH-1^low^ glioma patients (*n* = 50). **C** western blotting of L1CAM in LN229/T98G cells treated with PBS or serotonin (10 nM), and TPH-1 overexpressing LN229/T98G cells. **D** relative expression of L1CAM in LN229/T98G cells treated with scrambled and L1CAM siRNA, determined by qPCR. **E** cell proliferation of PBS cultured LN229/T98G (scrambled siRNA treatment), serotonin (10 nM) cultured LN229/T98G cells treated with scrambled and L1CAM siRNA. **F** relative migrating cells of PBS cultured LN229/T98G (scrambled siRNA treatment), serotonin (10 nM) cultured LN229/T98G cells treated with scrambled and L1CAM siRNA. **G** PBS cultured LN229/T98G (scrambled siRNA treatment) was collected, and serotonin (10 nM) cultured LN229/T98G cells were pre-treated with scrambled and L1CAM siRNA. Then cell apoptosis of LN229/T98G treated with PBS or TMZ (1 μg/ml, 48 h) were determined. **H** overall survival of glioma patients with high *L1CAM* (*n* = 49) and low *L1CAM* (*n* = 50) expression

To clarify the mechanism underlying serotonin promoting glioma development, we examined the mRNA expression profile of glioma cancer patients as shown in Fig. 1B. The top 30 overexpressed genes in the high TPH-1 group were identified (Fig. 3A). Notably, an increased mRNA expression of L1CAM was observed in the TPH-1 high glioma cancer tissues, and L1CAM reportedly serves as an L1 cell adhesion molecule, thereby participating in the process of cellular migration of tumor cells. Consistently, the GO enrichment analysis also indicated that the cell adhesion molecule signaling was involved in TPH-1 associated tumor progression (Fig. 3B). Therefore, we sought to examine the role of L1CAM in regulating glioma development. Thus, the expression of L1CAM was determined in THP1 overexpressing and serotonin-treated tumor cells. THP1 overexpression and serotonin treatment resulted in the upregulation of L1CAM in LN229 and T98G cells (Fig. 3C), which suggested a direct relationship between serotonin production and L1CAM signaling in glioma. To further confirm the role of L1CAM in the regulation of glioma progression, siRNA constructs were used to deplete L1CAM in LN229/T98G cells along with serotonin treatment (Fig. 3D). In these cells, L1CAM silencing significantly suppressed cellular proliferation (Fig. 3E) and migration (Fig. 3F) induced by serotonin. Consistently, the suppressive effects were also observed in cellular apoptosis analysis (Fig. 3G), suggesting that L1CAM played a role in glioma development. Additionally, L1CAM silencing also suppressed the pro-tumor effects induced due to TPH1 overexpression (Fig. S1C, D, and E). Furthermore, we observed that L1CAM expression was positively correlated with a poor prognosis of glioma patients (Fig. 3H). Taken together, these results suggested that serotonin facilitated glioma development by regulating L1CAM expression.

### Serotonin upregulates NF-κB expression through L1CAM signaling

**Fig. 4:**
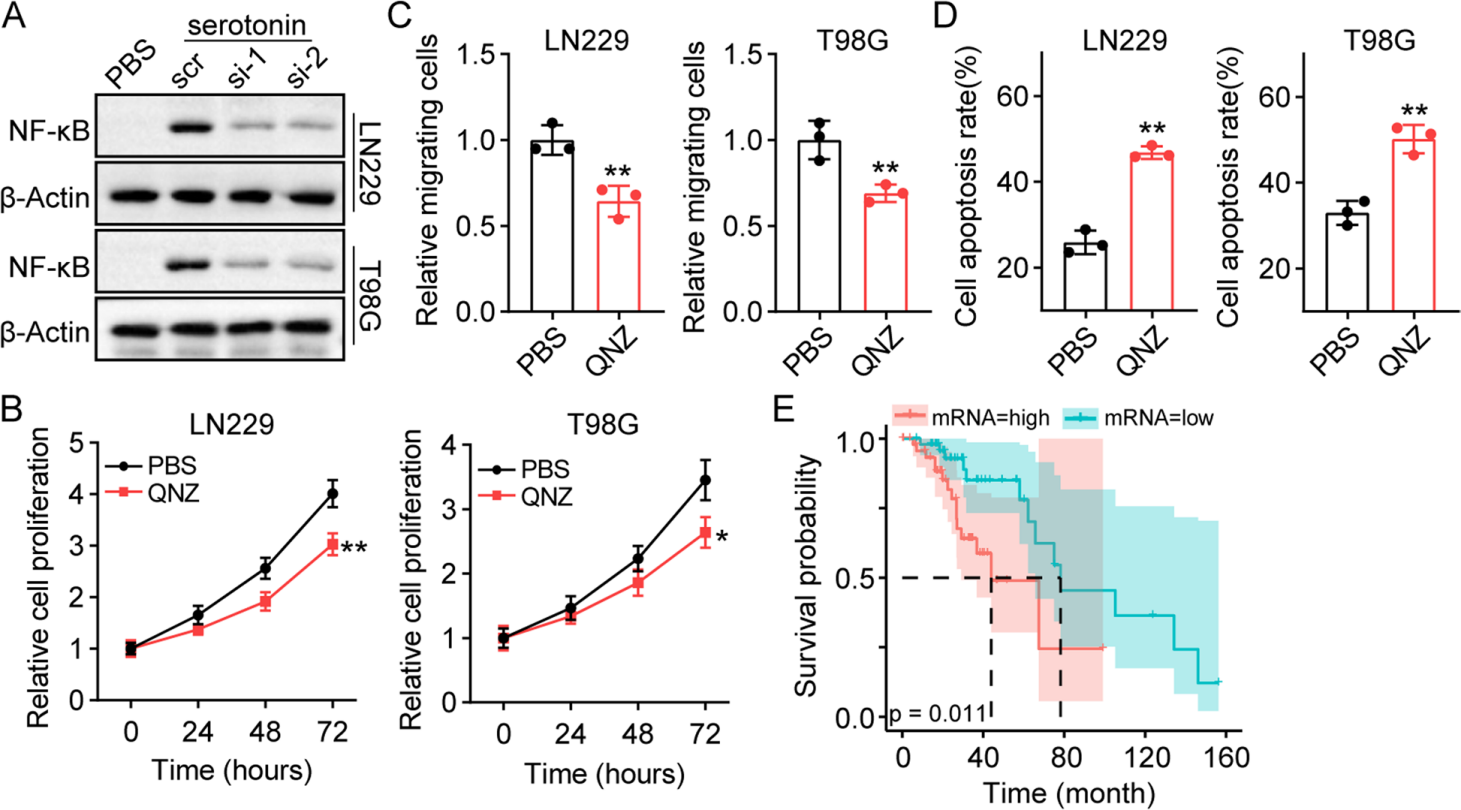
Serotonin upregulated NF-κB expression through LICAM signaling. **A** Western blotting of NF-κB in LN229/T98G cells, serotonin (10 nM) cultured LN229/T98G cells treated with scrambled and L1CAM siRNA. **B** Cell proliferation of serotonin (10 nM) cultured LN229/T98G cells treated with PBS and QNZ (10 nM). **C** Relative migrating cells of serotonin (10 nM) cultured LN229/T98G cells treated with PBS and QNZ (10 nM). **D** Serotonin (10 nM) cultured LN229/T98G cells were pre-treated with QNZ (10 nM, 48 h). Then cell apoptosis of LN229/T98G treated with PBS or TMZ (1 μg/ml, 48 h) were determined. **E** Overall survival of glioma patients with high *NFKB1* (*n* = 49) and low *NFKB1* (*n* = 50) expression

Next, we aimed to elucidate the mechanism underlying L1CAM-controlled tumor progression in glioma. As shown in Fig. 3B, the results of the GO enrichment analysis indicated that NF-κB signaling was substantially involved in TPH-1 associated biological activities. In fact, accumulating evidence suggests that L1CAM can induce constitutive NF-κB activation in pancreatic adenocarcinoma cells. The transcription factor, NF-κB, plays an essential role as a stressor in the cellular environment and controls the expression of various regulatory genes, that directly regulate cellular proliferation, migration, and drug resistance in several tumor types. To determine the role of NF-κB in glioma, we examined the protein expression of NF-κB in serotonin-treated LN229/T98G cells. Serotonin treatment increased the NF-κB levels in tumor cells, whereas L1CAM silencing suppressed the upregulation of NF-κB (Fig. 4A). Blockade of the NF-κB signaling cascade by QNZ efficiently suppressed the proliferative properties (Fig. 4B) and migratory potential (Fig. 4C) induced by serotonin. Similarly, QNZ treatment suppressed the TMZ resistance in serotonin-treated LN229 and T98G cells (Fig. 4D). These results showed that L1CAM upregulated NF-κB expression to regulate glioma development. Next, we examined the expressions of the downstream factors of NF-κB, including c-Myc, IL-8, IL-1β, VEGF, CDK2, and COX2. Serotonin treatment mediated the upregulation of c-Myc and IL-1β in LN229 and T98G cells, whereas QNZ treatment suppressed the gene upregulation induced by serotonin (Fig. S1F). Subsequently, we evaluated the influence of *NFKB1* expression on the prognosis of glioma patients. A poor overall survival was observed in patients with high *NFKB1* expression (Fig. 4E). Collectively, these results suggested that serotonin upregulated NF-κB expression through L1CAM signaling, thus resulting in tumor progression and a poor prognosis in glioma patients.

### Interruption of TPH-1 signals attenuates the aggressiveness of glioma in vivo

**Fig. 5:**
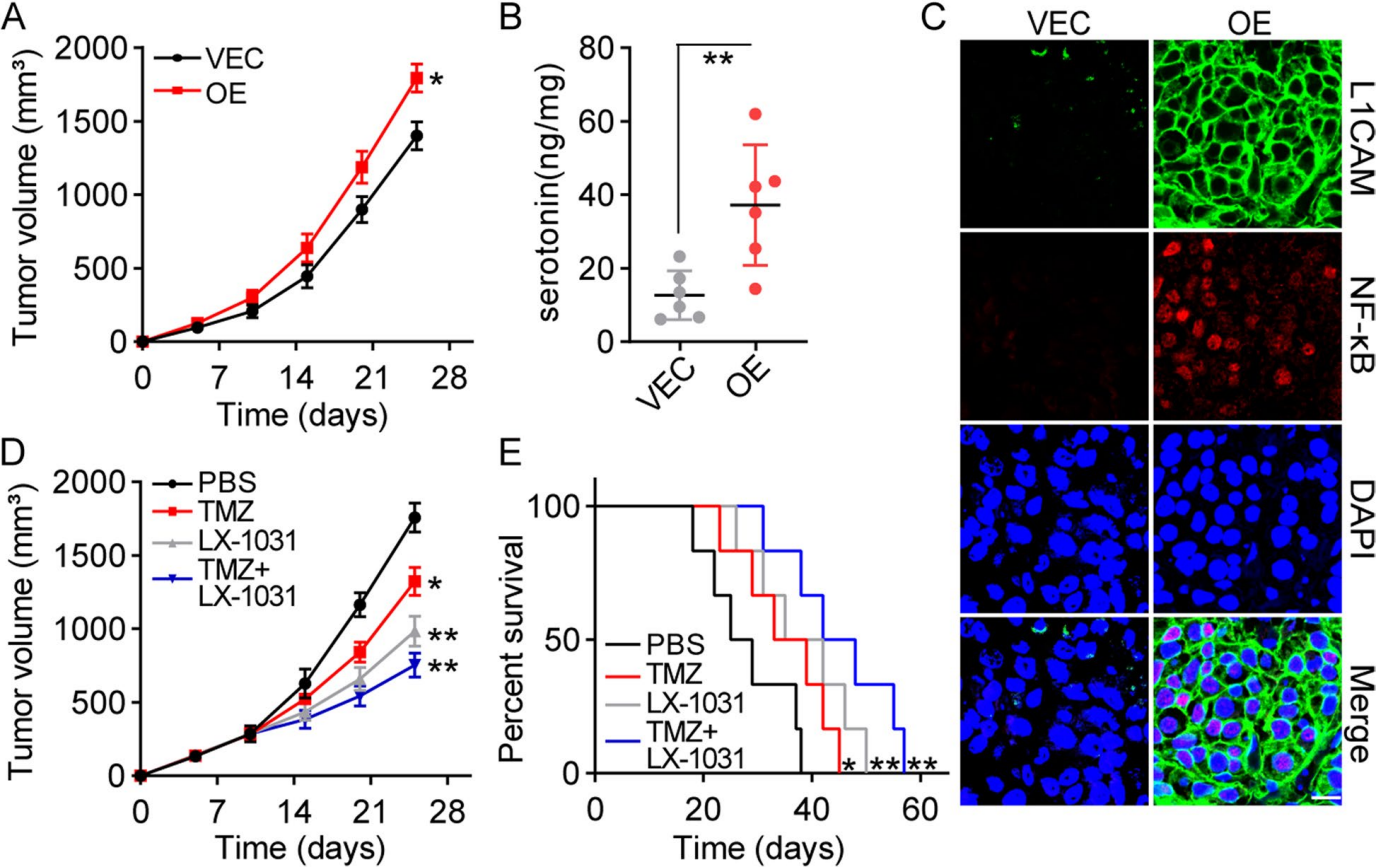
Interrupt of TPH-1 signals attenuated the aggressiveness of glioma in vivo. **A** LN229 (VEC) and TPH-1 overexpressing LN229 cells (OE) were subcutaneously injected into mice. The tumor volume was recorded. **B-C** The expression of serotonin (**B**), L1CAM and NF-κB (**C**) in tumor tissues from (**A**) was determined by Elisa assay or immunofluorescence. The scale bar was 25 μm. **D** Tumor volume of TPH-1 overexpressing LN229 bearing mice treated with PBS, TMZ, LX-1031 and TMZ combining LX-1031. **E** Survival time of TPH-1 overexpressing LN229 bearing mice treated with PBS, TMZ, LX-1031 and TMZ combining LX-1031

Our previous results suggested the role of TPH-1 and serotonin in promoting glioma cell proliferation. Therefore, we were interested in evaluating the influence of TPH-1 on glioma tumor growth in vivo. Thus, TPH-1 overexpressing LN229 cells were subcutaneously implanted into immunodeficient mice, and the tumor volumes were recorded. Consistent with in vitro results, a rapid tumor growth curve was observed in TPH-1 overexpressing LN229 bearing mice relative to the vec-LN229 bearing mice (Fig. 5A). In line with the high expression of TPH-1, higher protein levels of serotonin (Fig. 5B), L1CAM, and NF-κB (Fig. 5C) were found in TPH-1 overexpressing LN229 bearing mice (H&E staining in Fig. S1H). To retard the sustained tumor growth induced by TPH-1, we combined TMZ with the TPH-1 inhibitor, LX-1031, and treated the TPH-1 overexpressing LN229 bearing mice. Intriguingly, limited anti-cancerous effects were observed in the TMZ groups, consistent with our in vitro findings which suggested that TPH-1 promoted TMZ resistance in LN229 cells. Moreover, inhibition of TPH-1 by LX-1031 significantly suppressed the tumor growth and improved the outcomes of TMZ treatment (Fig. 5D). A similar result was recorded in the survival analysis for tumor-bearing mice (Fig. 5E). Those results suggested that TPH-1 may serve as a novel indicator for tumor diagnoses and provided innovative targets for glioma therapy.

## Discussion

Glioma, which exhibits strong invasiveness and a poor response to treatment, is generally difficult to cure due to the lack of effective pharmacological targets. Herein, we extended the prior work, and our findings underlined TPH-1 participation in regulating the proliferative properties, migratory capacity, and chemotherapeutic sensitivity of glioma cells. Moreover, TPH-1 blockade produced a substantial suppression of tumor growth in a mouse model of glioma when administered in conjunction with chemotherapeutic agents. These data may broaden the understanding of the complexity of glioma and aid in the development of therapeutic strategies.

Recent advances in energy metabolism link amino acid metabolism to the malignant etiologies of cancer [23]. Trp catabolism is among the most researched topics, and several enzymes, as well as metabolites involved in tryptophan degradation, contribute to the aggressive traits of tumor cells [24]. Apart from the Kyn pathway, accumulating evidence shows the carcinogenic properties of the serotonergic pathway [25, 26]. During tumor progression, breast cancer tissues display elevated TPH-1 expression corresponding to enhanced serotonin synthesis [27]. Gianfranco et al. report that serotonin treatment causes a significant increase in the proliferative capacity of the cholangiocarcinoma cells and this change can be reversed by TPH-1 blockade [28]. Our study focused on the role of TPH-1 in glioma and the findings indicated that TPH-1 hydroxylated Trp led to serotonin production, thereby driving robust tumor growth and progression. Moreover, our data linked TPH-1 to the chemotherapeutic sensitivity of glioma, demonstrating that TMZ resistance was aggravated in the LN229/T98G cells overexpressing TPH-1. To further investigate the clinical implications of the serotonergic pathway, we performed analysis using TCGA dataset, which suggested that a high TPH-1 expression was associated with a poor prognosis in glioma patients. Therefore, it was imperative to disentangle the mechanisms underlying TPH-1-mediated regulation of glioma development.

L1CAM, a transmembrane glycoprotein of the immunoglobulin superfamily, was originally described in the nervous system, whereby it plays a role in brain development and functions [29]. Subsequent research attests to the presence of L1CAM in several cancer cell types, and a high L1CAM expression correlates with advanced tumor stages and grave prognoses [30]. L1CAM endows cancer cells with enhanced tumorigenic properties and motility, which can be reversed by gene silencing as well as antibodies [31]. L1CAM has been exploited as a diagnostic marker and more importantly as a promising therapeutic target for the treatment of malignancies. The control of L1CAM expression in cancer has therefore drawn widespread research attention. Previous studies indicate that L1CAM, situated specifically at the invasive front of tumor tissues, serves as a target for activation by β-catenin-TCF signaling in colorectal cancer [32]. Subsequent studies have provided evidence that the transforming growth factor-beta1 (TGF-β1) augments L1CAM expression in pancreatic and endometrial cancer cells, which is dependent on Slug, a transcription factor that modulates epithelial-mesenchymal transition [33–35]. In glioma, shutting down of L1CAM expression causes complete cessation of cellular migration [36]. In line with previous findings, we confirmed the growth-promoting effects of L1CAM and additionally found that L1CAM knockdown increased the chemotherapeutic sensitivity of LN229 and T98G cells. Importantly, we have highlighted the involvement of L1CAM in TPH-1 mediated glioma progression, as indicated by the upregulation of L1CAM in THP1 overexpressing glioma cells. Moreover, clinical data analysis demonstrated that L1CAM expression was a risk factor for glioma patients, with higher values pertaining to a poor prognosis. Mechanistically, L1CAM employed several downstream pathways through its interaction with different cell surface receptors. Steve et al. report that ectopic L1CAM expression in 3 T3 and K1735-C11 cells induces sustained extracellular signal-regulated kinase (ERK) activation, which may require the participation of growth factor [37]. L1CAM can also be cleaved by the metalloproteinases, ADAM10 and ADAM17, which further results in the release of a 200 kDa soluble ectodomain fulfilling diverse functions in both tumor and immune cells [38]. In the present study, we found that serotonin treatment could significantly elevate LICAM expression, which further contributed to the activation of NF-κB, a crucial player in human neoplasms as it empowers several key attributes of cancer cells. Consistently, GO enrichment analysis indicated the engagement of NF-κB signaling in TPH-1-related biological activities. Therefore, targeting TPH-1 may be a feasible modality for curing glioma. Our in vivo experiments demonstrated that LX-1031 co-operated with TMZ to suppress tumor growth and prolong survival, thus eventually resulting in favorable outcomes in glioma-bearing mice. Based on the above results, our findings demonstrated that TPH-1 contributed to glioma development through the serotonin/L1CAM/NF-κB signaling pathway and offered possibilities for the application of TPH-1 inhibitors in glioma therapy.

In light of the limitations to previous studies, herein, we highlight the relevance of TPH-1 in glioma advancement. We showed that (1) TPH-1 played a stimulatory function for glioma cell proliferation, motility, and drug resistance. (2) The tumor-promoting effects of TPH-1 were dependent on the serotonin/L1CAM/NF-κB signaling pathway. (3) The cessation of glioma growth could be accomplished by TMZ in combination with an L1CAM inhibitor, hinting at a potential target for therapeutic intervention. (4) The levels of TPH-1, L1CAM, and NF-κB in tumor tissues may serve as potential biomarkers for monitoring glioma progression and predicting prognoses.

## Conclusion

In conclusion, we demonstrated that TPH-1 functioned through the serotonin/L1CAM/NF-κB pathway, thus facilitating glioma cell proliferation, migration, and chemoresistance. Targeting TPH-1 may open new avenues for the clinical treatment of glioma.

## Supplementary Information

**Additional file 1.**

**Additional file 2.**

## Data Availability

The datasets generated and/or analysed during the current study are available on https://figshare.com/s/829e7f54b49e7f6d3713.

## Abbreviations

TCGA: The Cancer Genome Atlas
TPH-1: Tryptophan hydroxylase 1
TMZ: Temozolomide
L1CAM: L1-cell adhesion molecule
Kyn: Kynurenine
IDO: Indoleamine-2, 3-dioxygenase
TDO: Tryptophan-2, 3-dioxygenase
TNBC: Triple-negative breast cancer
TGF-β1: Transforming growth factor-beta1
ERK: Extracellular signal-regulated kinase
