# Supplementary material for: Tryptophan hydroxylase 1 drives glioma progression by modulating the serotonin/L1CAM/NF-κB signaling pathway
Source: BMC Cancer. 2022 Apr 26;22:457. doi: 10.1186/s12885-022-09569-2 (PMC9044587; doi:10.1186/s12885-022-09569-2)
Supplement: Supplementary file 1 — Additional file 1. [file 12885_2022_9569_MOESM1_ESM.docx]

­Supplementary information


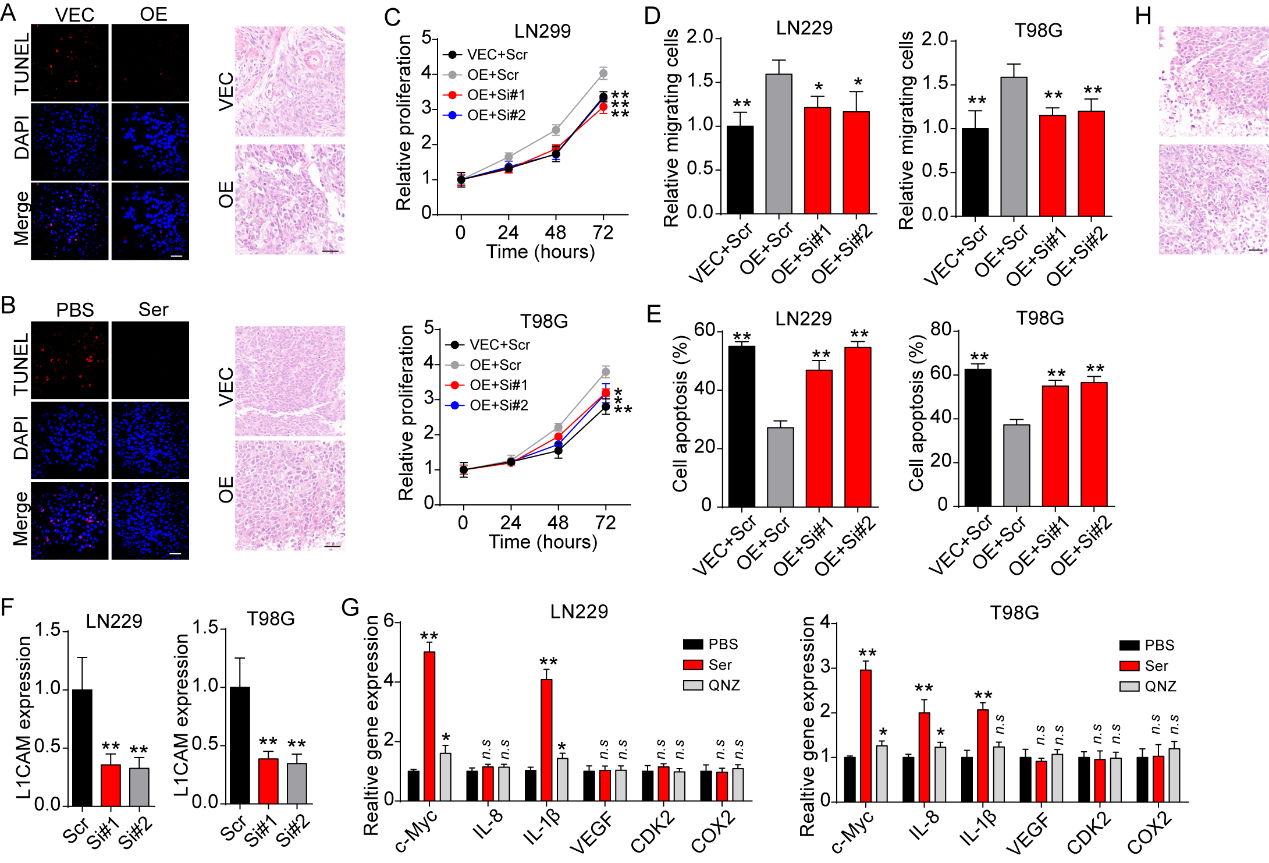


Supplementary figure 1

A, TUNEL assay of tumor tissues from vector or TPH1 overexpression LN229-bearing mice, treated with TMZ. The scale bar was 50 μm. Tumor tissues were validated using H&E staining. The scale bar was 50 μm. B, TUNEL assay of tumor tissues from LN229-bearing mice, treated with PBS or serotonin, following with TMZ therapy. The scale bar was 50 μm. C, cell proliferation of vector LN229/T98G (Scr siRNA treatment), TPH1 overexpression LN229/T98G cells treated with Scr or L1CAM siRNA. D, relative migrating cells of vector LN229/T98G (Scr RNA treatment), TPH1 overexpression LN229/T98G cells treated with Scr or L1CAM siRNA. E, vector LN229/T98G (Scr RNA treatment), TPH1 overexpression LN229/T98G cells treated with Scr or L1CAM siRNA were collected. Then cell apoptosis of LN229/T98G treated with TMZ (1 μg/ml, 48 hours) were determined. F, relative expression of *L1CAM* was determined in vector or L1CAM silenced LN229/T98G cells by quantitative PCR. G, relative expression of *c-Myc, IL-8, IL-1β, VEGF CDK2* and *COX2* was determined in LN229/T98G cells treated with PBS, serotonin (10 nM) or serotonin combining QNZ (10 nM) by quantitative PCR. H, H&E staining of tumor tissues from vector or TPH1 overexpressing LN229-bearing mice. The scale bar was 50 μm.
