# Supplementary figures and images for: Tryptophan hydroxylase 1 drives glioma progression by modulating the serotonin/L1CAM/NF-κB signaling pathway
Source: BMC Cancer. 2022 Apr 26;22:457. doi: 10.1186/s12885-022-09569-2 (PMC9044587; doi:10.1186/s12885-022-09569-2)

Fig. 1C actin


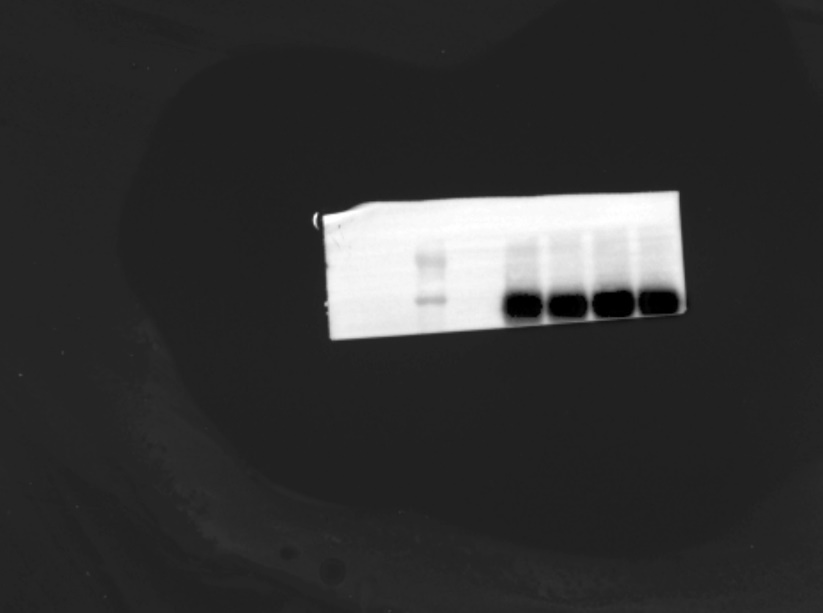


**45kd**

Fig. 1C TPH1


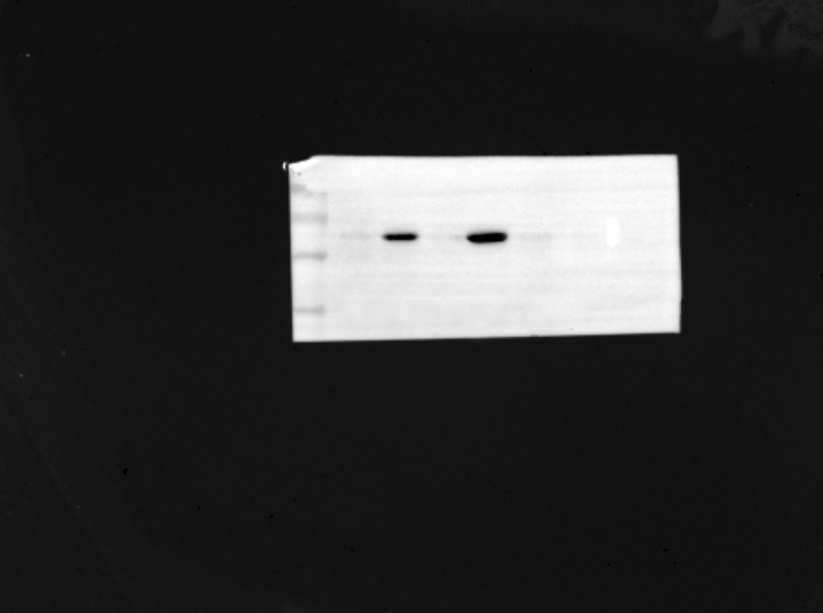


**60kd**

**45kd**

Fig. 3C actin


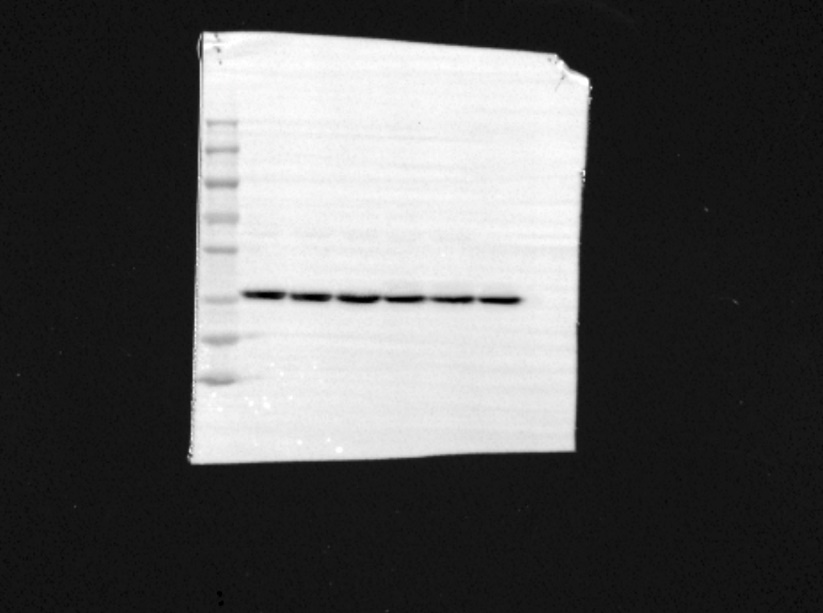


**45kd**

Fig. 2C


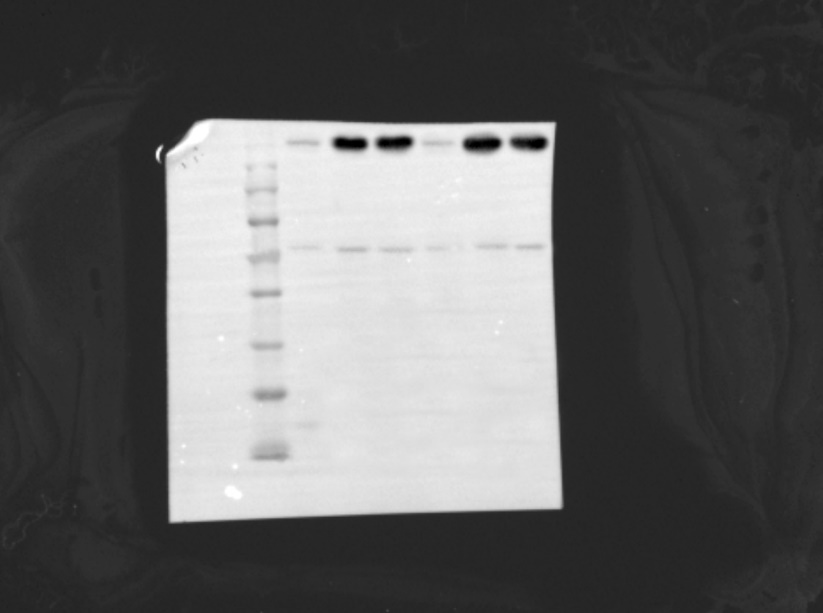


**180kd**

Fig. 4A actin 1


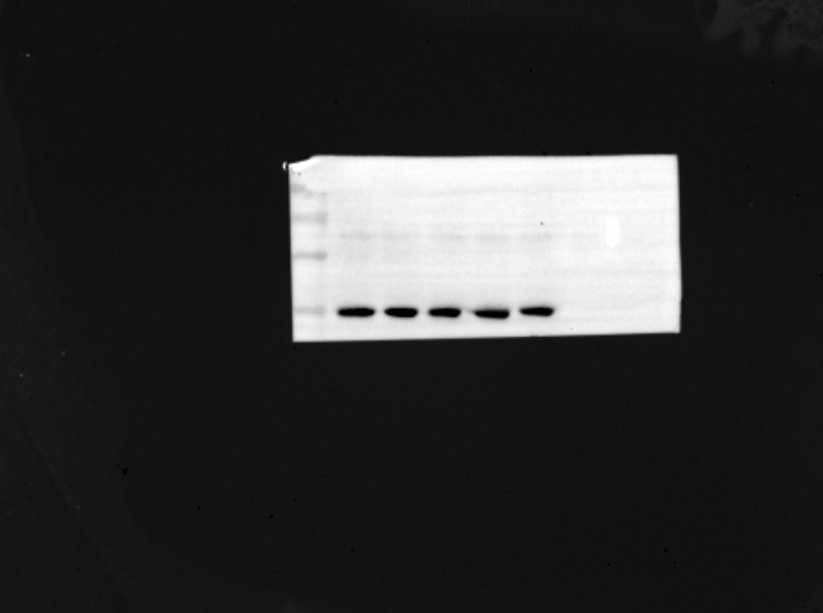


**45kd**

Fig. 4A actin 2


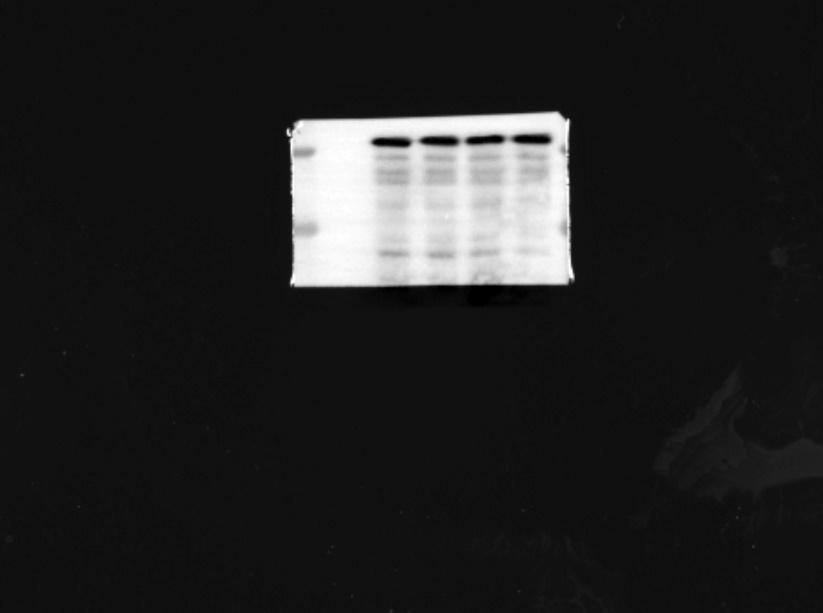


**45kd**

­­

Fig. 4A NFKB


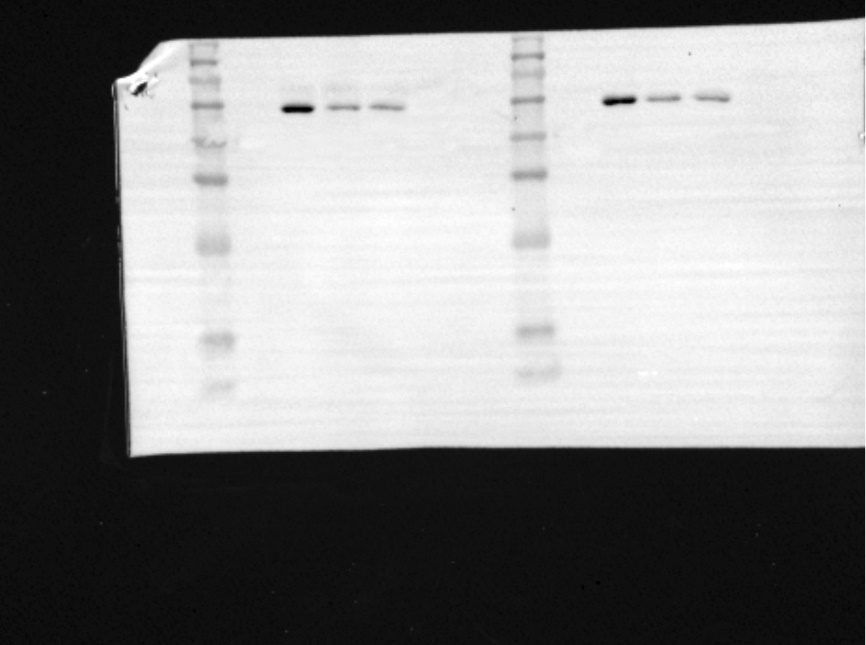


**75kd**

Supplement: Supplementary file 2 — Additional file 2. [file 12885_2022_9569_MOESM2_ESM.docx]
